# Restrictive Ventilatory Impairment Due to Severe Scoliosis with a Large Cobb Angle

**DOI:** 10.31662/jmaj.2023-0002

**Published:** 2023-09-27

**Authors:** Yuya Ando, Yosuke Ono, Sachiko Ono, Yuji Tanaka

**Affiliations:** 1 Department of General Medicine, National Defense Medical College, Saitama, Japan; 2 Department of Internal Medicine, Self-Defense Forces Central Hospital, Tokyo, Japan; 3 Department of Family Medicine, Tokyo Medical and Dental University, Tokyo, Japan; 4 Department of Eat-loss Medicine, The University of Tokyo, Tokyo, Japan

**Keywords:** scoliosis, Cobb angle, restrictive ventilatory impairment, respiratory failure, pulmonary rehabilitation

A 43-year-old woman presented with increasing shortness of breath over a one year period and had a history of scoliosis diagnosed at the age of 10; however, she had repeatedly refused surgical interventions or prosthetics. She appeared pale during physical examination, and her respiratory rate was 48/min with oxygen saturation of 58% while breathing room air. Arterial blood gas analysis at 2 L/min supplemental oxygen showed pH of 7.33, PaCO_2_ of 63.7 Torr, PaO_2_ of 75.3 Torr, and HCO_3_^−^ of 32.8 mmol/L. Moreover, blood tests revealed iron deficiency anemia (hemoglobin, 7.8 g/dL) and elevated brain natriuretic peptide (268.9 pg/mL), and pulmonary function tests revealed severe restrictive ventilatory impairment (forced vital capacity percentile, 21.4%). Furthermore, chest radiography showed severe scoliosis (Cobb angle, 116°) (Figure 1), and three-dimensional chest computed tomography confirmed a reduction in right lung field (Figure 2). Consequently, she was diagnosed with hypercapnic respiratory failure secondary to untreated scoliosis and heart failure exacerbated by anemia. Her condition improved after implementation of a bilevel positive airway pressure device and administration of oral iron supplements. After discharge, she received home oxygen therapy and regular pulmonary rehabilitation including thoracic range of motion exercises, 20-minute walking, and light load resistance training. During 8 years of follow-up, she did not report any recurrence. Literature has reported that a Cobb angle >110° and vital capacity <45% are highly related to the development of respiratory failure in skeletally mature patients ^(1)^, and clinicians should be well aware of this risk.

**Figure 1.**
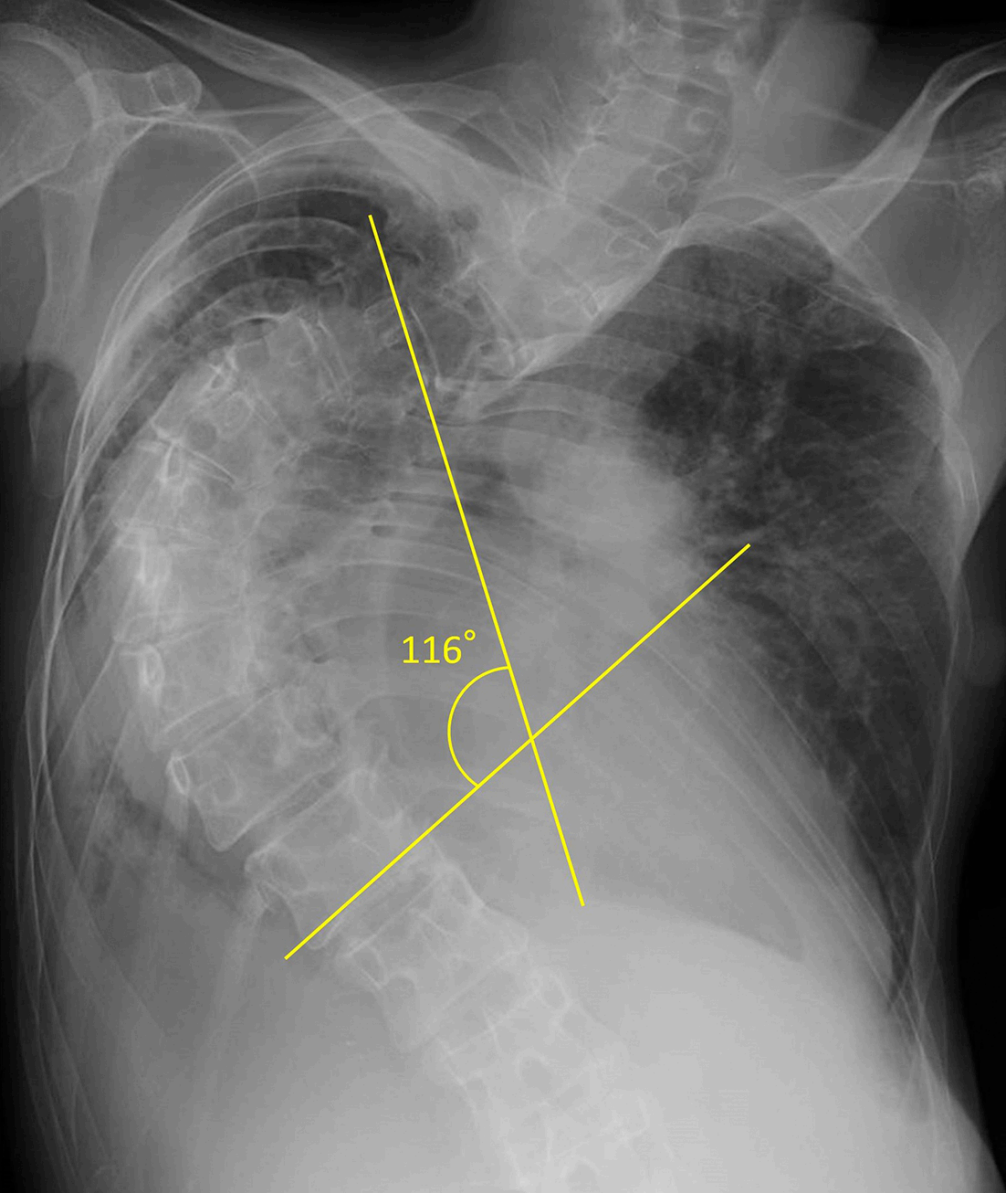
Chest radiography depicting severe scoliosis (Cobb angle, 116°).

**Figure 2.**
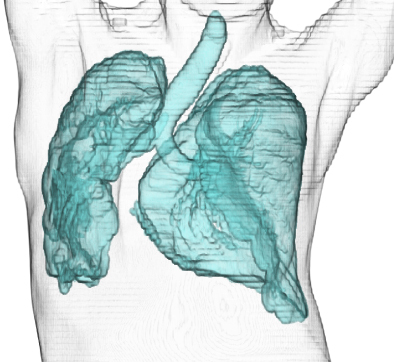
Three-dimensional chest computed tomography image showing the entire lung structure and reduction in lung volumes.

## Article Information

### Conflicts of Interest

None

### Sources of Funding

This work was supported by the Japan Medical Education Foundation (https://www.jmef.or.jp/).

### Author Contributions

YA wrote the first draft of the manuscript, and YO and SO revised the manuscript. Furthermore, YO and YT contributed to the patient care, and YT organized the manuscript.

### Informed Consent

The patient provided written informed consent for the publication of the manuscript and clinical images.

### Approval by Institutional Review Board (IRB)

This study did not require IRB approval.
